# Long-Term Arteriovenous Access Clinical Patency Following Successful Thrombolysis: A Single-Centre Experience

**DOI:** 10.7759/cureus.81779

**Published:** 2025-04-06

**Authors:** Maysoon ElKhawad, Baljeet Dhillon, Tariq Ali, Philip C Bennett

**Affiliations:** 1 Vascular Surgery, Norfolk and Norwich University Hospitals National Health Service (NHS) Foundation Trust, Norwich, GBR; 2 Radiology, Norfolk and Norwich University Hospitals National Health Service (NHS) Foundation Trust, Norwich, GBR; 3 Interventional Radiology, Norfolk and Norwich University Hospitals National Health Service (NHS) Foundation Trust, Norwich, GBR

**Keywords:** arteriovenous access, av fistula, av graft, clinical patency, thrombolysis

## Abstract

Introduction

There is a paucity of data regarding the long-term outcomes following thrombolysis of arteriovenous (AV) access for dialysis. The aim was to determine the technical and clinical success following thrombolysis of a thrombosed AV access and the long-term clinical patency and access survival in our institution.

Methods

Retrospective identification of all patients undergoing thrombolysis of an AV access, AV fistula (AVF), or graft (AVG) over eight years at a single institution. Patient characteristics, access type, type of thrombolysis, and fistula life were recorded. Data were censored for death, transplantation, and loss to follow-up.

Results

Ninety-eight vascular accesses (79 AVF and 19 AVG) in 94 patients underwent thrombolysis during the study period. Fifty-three (56.4%) were male with a median (interquartile range [IQR]) age of 66 (53-74) years. Immediate technical and clinical success were 82% and 75%, respectively. Clinical patency following clinically successful thrombolysis at three, six, and 12 months was 64.5%, 60%, and 45.9%, respectively. At two and three years, clinical patency was 21.3% and 7.9%, respectively. AVFs were older than AVGs, 96 (44-192) vs. 20 (12-76) months, p<0.0001 at the time of thrombolysis. 21% of AV accesses that had successful thrombolysis and restoration of clinical patency re-presented with thrombosis after a median (IQR) of 185 (93.5-358) days and underwent further thrombolysis. AVGs (47.4%) were more likely to have re-thrombosed after successful thrombolysis than AVFs (15.2%), p=0.002. Kaplan-Meier survival analysis demonstrated that AVFs remained patent for longer than AV grafts (p=0.023) following successful thrombolysis. While in the short term, clinical patency was re-established, overall, there was no AV access survival advantage (p=0.085) over those that only required one episode of thrombolysis to restore access patency.

Conclusions

Endovascular strategies for dealing with thrombosed access are clinically important in allowing haemodialysis to continue. However, the clinical patency rates, while good in the short term, fail to be sustained long term. We propose that an episode of AV access thrombosis and successful thrombolysis should prompt a review of previous access-related interventions, age of AV access, and planning for alternative AV access options, be it new AV access formation, transplant, or long-term catheter placement.

## Introduction

Thrombosis of arteriovenous (AV) access is a serious and often terminal event for failing access. As well as low AV access flow rates, thrombosis accounts for the majority of AV access losses [1], and represents 65% to 85% of all cases of access abandonments [2]. While the precipitating cause of the terminal event is often apparent, such as venous outflow stenoses or low flow in the case of an arteriovenous graft (AVG), a patient may present with a thrombosis without any obvious mechanism [2,3].

Several techniques, including image-guided surgery, percutaneous intervention, or a combination of both [4], can be used to restore dysfunctional or thrombosed access, with the choice of treatment depending on availability, centre experience, and operator preference. The latest Kidney Disease Outcomes Quality Initiative (KDOQI) guidelines advocate both surgical and endovascular thrombectomy procedures, though there is limited data comparing the two [2]. A meta-analysis comparing surgical thrombectomy to endovascular therapy for thrombosed AVGs found outcomes to be comparable [5]. Observational studies on the treatment of thrombosed arteriovenous fistulas (AVFs) were identified and also showed similar primary outcomes of surgical and endovascular interventions [6]. Given that vein stenosis is one of the most common underlying causes, the endovascular approach has gained popularity as it allows both thrombectomy and treatment of any underlying lesion in the same sitting. Endovascular interventions have therefore become the first-line treatment for dialysis access thrombosis and dysfunction, when possible, with more than two-thirds occurring in the outpatient setting [7,8]. However, identifying stenotic lesions and treating them preemptively has not been shown to prevent re-thrombosis [9].

Percutaneous or open salvage attempts of thrombosed access should be performed within 24 to 48 hours, whenever achievable [7]. However, there is evidence that thrombolysis can be successful even after a significant delay [10].

The existing literature mainly focuses on immediate technical success. Although immediate success in providing at least one effective dialysis session after the procedure can be achieved in 80% to 95% of thrombosed AVGs and AVFs [11,12], long-term patency after thrombectomy remains elusive, with reported rates of 25% to 50% at six months and 10% to 20% at one year [12,13].

Given the consequences of vascular access thrombosis and potential risks of thrombolysis on the patient, we sought to look at outcomes of thrombolysis in our institution to determine if there is any long-term benefit to the patient in AV access salvage and if there are any predictors of access survival following clinically successful thrombolysis. This article was previously presented at the Vascular Access Society of Great Britain and Ireland in 2020.

## Materials and methods

Our centre has a dialysis population of approximately 230 patients, and the vascular unit undertakes 80-90 vascular access procedures per year. This was a retrospective study of all patients undergoing thrombolysis in the radiology department at our institution. Patients were identified from a radiology database, and all procedures performed within an eight-year period were included. Patients were screened for potential contraindications to thrombolysis prior to the procedure. Patient characteristics were sought from a local renal database (EMedrenal v2.3.2, MEDIQUAL H.I.). 

Patients presenting to our hospital with a clinically thrombosed AVF/AVG or a failure of cannulation for haemodialysis underwent a confirmatory ultrasound in the vascular studies department. Fistulas that had not thrombosed did not undergo thrombolysis. Early primary failure of a fistula (less than six weeks) was not considered for thrombolysis, but preoperative imaging and operation notes were reviewed, and if appropriate, these patients were taken to the theater for surgical thrombectomy. A decision on whether to proceed with thrombolysis was based on clinical judgement and made jointly by the renal and vascular consultants on call and the dialysis access specialist nurse. This decision was based upon patient comorbidities, assumed length of time that the fistula had been thrombosed, availability of IRU and critical care beds, whether the patient had any future access options, and whether it was a simple or complex AVF or graft. All patients were admitted under the care of the renal team on the renal ward prior to thrombolysis and were subsequently transferred under the care of the vascular team throughout the duration of their thrombolysis and stayed in the critical care unit.

Procedure

All procedures were performed under local anaesthesia. Ultrasound-guided access was obtained, which was either unidirectional or in opposing directions to traverse the occluded segment of the vein/graft. Typically, 5 or 6 Fr sheaths were used for access. The thrombus was crossed with a combination of catheter and hydrophilic wire into a patent vein segment. These were then exchanged for a thrombolysis catheter with multiple side holes to infuse thrombolytic agents (e.g., Alteplase). Thrombolysis was usually infused at a rate of 1 mg/hour (split if using crossing catheters), alongside a heparin infusion of 400 units/hour via the sheath (again, split if using crossing access). Our local policy is to include antibiotic cover using teicoplanin while the sheaths are in place. The patients were usually brought back to the interventional radiology suite the following day for repeat venography and definitive treatment of any causative lesions. For grafts, we opted for mechanical thrombectomy instead (e.g., AngioJet™, Boston Scientific, Marlborough, MA, USA) or if there was a perforation of the fistula vein (which would preclude chemical thrombolysis). Treatment of the causative lesions would usually involve plain balloon angioplasty, with occasional use of cutting balloons or stents for very recalcitrant stenoses. The use of drug-eluting technology is low at our institution.

Technical success was considered when spontaneous flow through the fistula vein was re-established. Clinical success would depend upon being able to undergo successful haemodialysis following thrombolysis.

The primary endpoint was AV access survival, denoted by an absence of fistula thrombosis and abandonment or unsuccessful restoration of flow to adequately support haemodialysis at the end of the study period.

Clinical patency was reported as a functioning fistula to adequately meet the haemodialysis needs of the patient, regardless of subsequent access interventions. Re-thrombosis of an AV access was confirmed on departmental ultrasound.

Statistical analysis

Data were censored for death and transplantation. Continuous variables were assessed using the Kolmogorov-Smirnov test for normality distribution. Pre-dialysis patients and patients with a functional transplant were excluded from the study. Data for continuous variables were expressed as mean ± standard deviation (SD). Where this was not normally distributed, it is expressed as median (interquartile range, IQR). Kaplan-Meier plots were used to analyse survival outcomes. Cox proportion hazard modelling was used to look at predictors of outcomes in thrombolysis. Data was analysed using Minitab Statistical Software (State Cal, USA).

## Results

Baseline demographics are summarized in Table 1.

**Table 1:** Patient demographics *Values expressed as median (inter-quartile range). AV: Arteriovenous, t-PA: Alteplase

	Thrombosed AV Access (n=98)
Female, no. (%)	45 (46%)
Male, no. (%)	53 (54%)
Age (years)*	66 (53-74)
AV Access age at presentation (months)*	84 (36-184)
Thrombolysis type (%)	
t-PA	89 (91%)
AngioJet	9 (9%)
Fistula type (%)	
Radio-cephalic	21 (22%)
Brachio-cephalic	45 (46%)
Brachio-basilic	13 (13%)
Arm graft	14 (14%)
Leg loop graft	5 (5%)

Ninety-eight vascular accesses (79 AVF and 19 AVG) in 94 patients underwent thrombolysis during the study period. Fifty-three (56.4%) were male with a median age of 66 (53-74) years. Forty-six (46.9%) patients were taking antiplatelets or anticoagulants at the time of access thrombosis, with no difference between AVG and AVF patients. Fifty (51.1%) patients died over the follow-up period. Fistulas undergoing thrombolysis were older than grafts, 96 (44-192) vs. 20 (12-76) months, p<0.0001. 57 (57%) AV accesses had undergone previous fistuloplasty for a US-detected >50% access stenosis, and six (four AV fistulas, two AV grafts) had successful thrombolysis for a thrombosed AV access prior to the study period.

Technical and clinical success

Eighty-nine (91%) AV accesses underwent catheter-directed thrombolysis with alteplase, and eight (nine%) underwent mechanical thrombectomy with the AngioJet™ system. Eighty (82%) AV accesses had technical success following thrombolysis, and clinical success was achieved in 74 (75%) accesses. All but one patient underwent ultrasound surveillance six weeks after clinical success, which demonstrated radiological patency with no recurrent stenoses. Clinical patency following clinically successful thrombolysis at three, six, and 12 months was 64.5%, 60%, and 45.9%, respectively. At two and three years, clinical patency was 21.3% and 7.9%, respectively.

**Figure 1:**
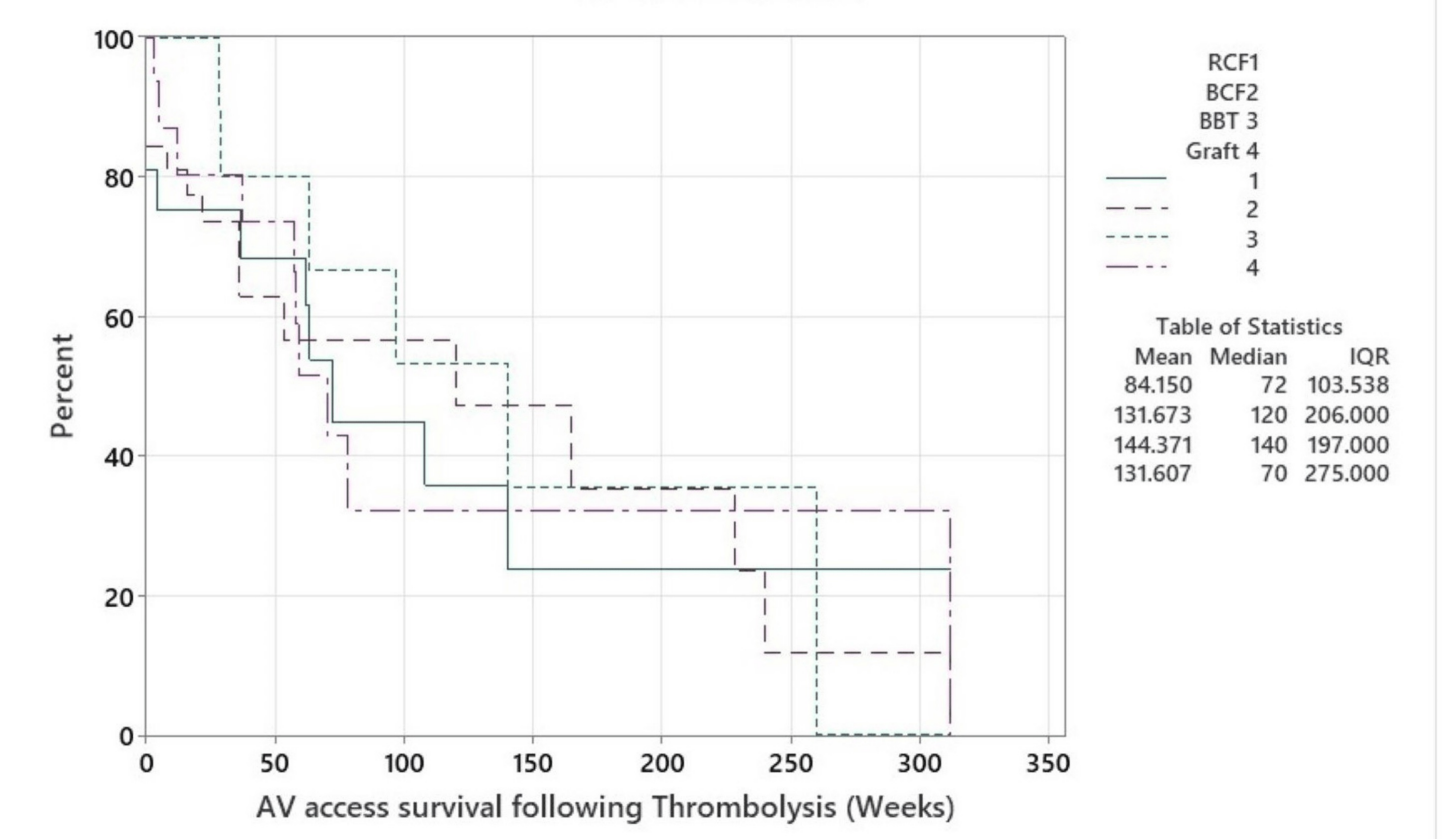
AV access survival following thrombolysis

The median AV access survival of vascular access after thrombolysis was 29 weeks (1-71 weeks). Kaplan-Meier analysis of the AV access survival was undertaken by fistula type (Figure 1) and did not demonstrate any differences by access type. 

Cox proportion hazard modelling was used to investigate variables associated with AV access survival on univariable analysis (Table 2).

**Table 2:** Univariable predictors of AV access survival following thrombolysis

Variable	RR	95% CI	p-value
Fistula Age	1	0.99-1.00	0.77
Graft vs. fistula	1.3	0.55-3.06	0.548
Previous fistuloplasty	1.8	0.76-4.28	0.185
Repeat thrombolysis	1	0.41-2.35	0.959
Antiplatelet usage	1.7	0.74-3.83	0.67

No variables were found to be significantly associated with access survival on univariable analysis overall or in fistulas alone. However, older grafts had better six-month primary patency. Median age of grafts remaining patent at six months: 119.2 (67.4-206.2) weeks vs. 42 (13.1-106.9) weeks for those thrombosed by six months. However, no differences in one-year patency were identified by graft age.

Recurrent thrombosis outcomes

A total of 21% of AV accesses that had successful thrombolysis and restoration of clinical patency re-presented with thrombosis after a median of 185 (93.5-358) days and underwent further thrombolysis. AVGs (47.4%) were more likely to have re-thrombosed after successful thrombolysis than AVFs (15.2%), p=0.002.

**Figure 2:**
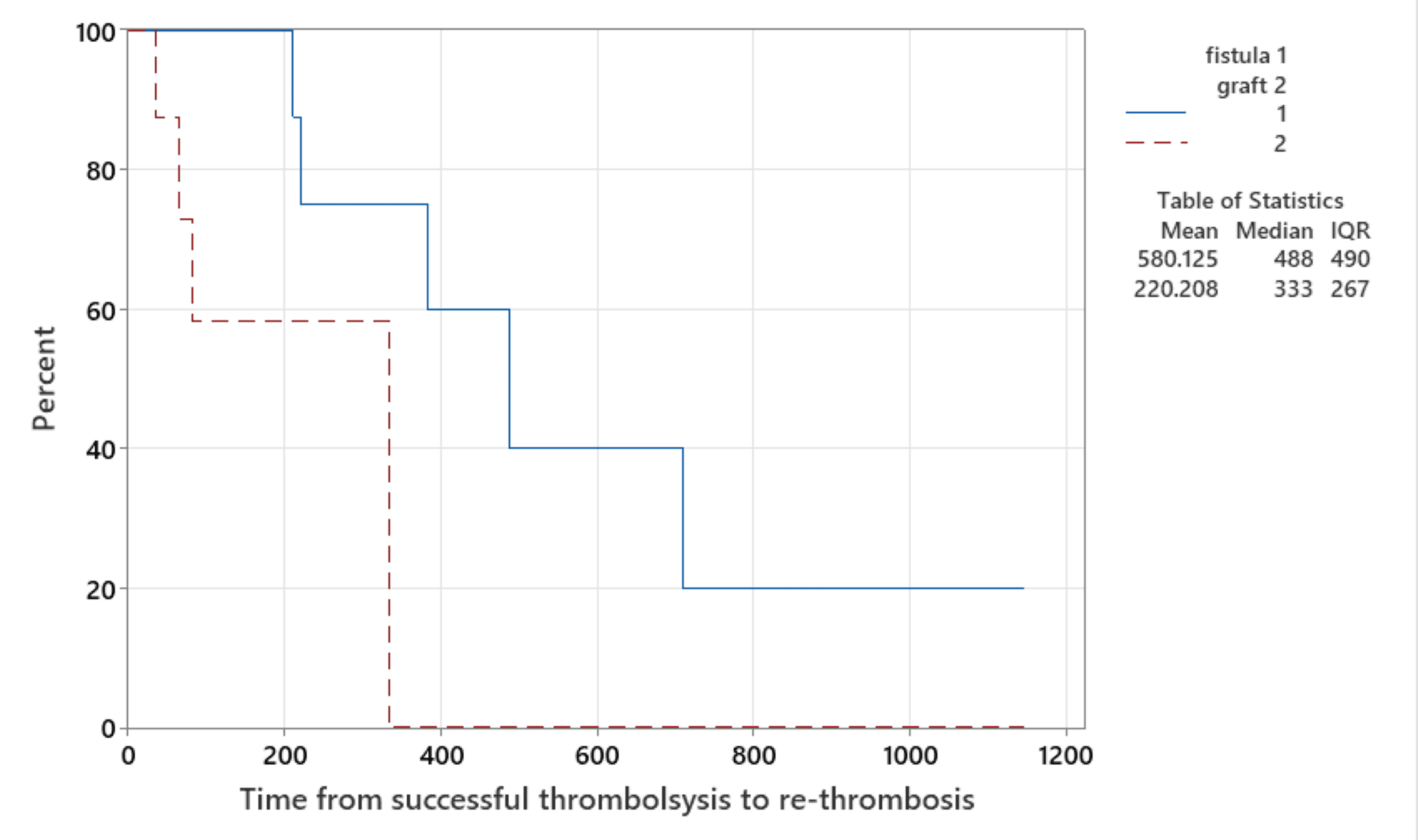
Survival plot of time from successful thrombolysis to re-thrombosis

**Figure 3:**
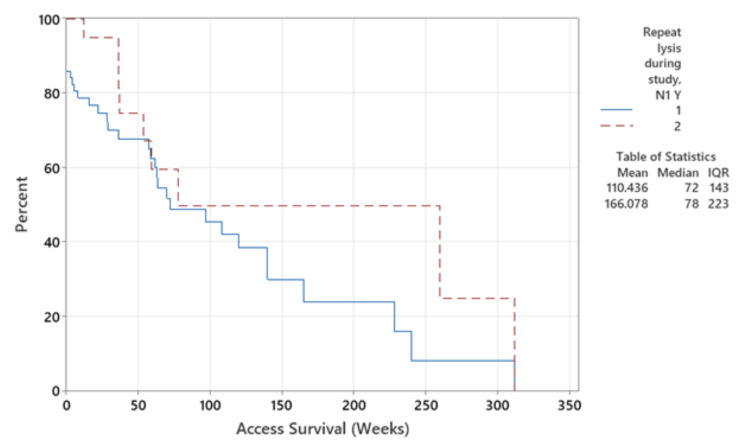
AV access survival following single or repeat episode of thrombolysis

Kaplan-Meier survival analysis (Figure 2) demonstrated AV fistulas remained patent for longer than AV grafts (p=0.023). While in the short term, clinical patency was re-established, overall, there was no AV access survival advantage (p=0.085 overall, p=0.074 in AVF alone, and p=0.715 in AVG alone) over those that only required one episode of thrombolysis to restore access patency (Figure 3).

## Discussion

AV access thrombosis accounts for 65-85% of access loss, with the associated increase in healthcare expenditures for patients with end-stage kidney disease (ESKD) [8,14]. In addition to increased morbidity, access-related thrombosis is the most burdensome complication to patients [15]. AV access thrombosis presents a clinical dilemma for patients undergoing renal dialysis. This leads to missed or delayed haemodialysis, a potential hospital admission, and placement of a temporary dialysis catheter, with the ensuing risk of catheter-related complications. Attempts at AV access salvage are, therefore, recommended by the European Society of Vascular Surgery and KDOQI [2,16]. The exact strategy of AV access salvage has sparser evidence, with guidelines acknowledging the paucity of evidence and recommending that salvage of vascular access be based on patient and local factors.

KDOQI guidelines, based upon expert opinion, consider it reasonable that management of each episode of AV access thrombosis is at the operator’s/clinician’s best judgement and discretion and involves the consideration of the patient’s dialysis access succession plan that is consistent with the ESKD life plan, given the compromised AV access patency after either endovascular or surgical treatment [2]. The preferred salvage process for patients presenting with thrombosis of an established AV access that has been previously used for haemodialysis and is more than six weeks old, in our institution, is endovascular so that any underlying stenotic lesions can be treated at the same time.

Much of the evidence for the management of fistula thrombosis is observational and mostly short- to medium-term outcomes [6]. A meta-analysis comparing surgical and endovascular salvage of thrombosed AV grafts showed outcomes to be comparable [5]. Technical success after a single attempt at fistula thrombolysis or thrombectomy is in the region of 90% and 80% in acutely thrombosed AVGs and AVFs, respectively [12,17,18]. However, long-term clinical patency after thrombectomy remains elusive, with reported rates of 25% to 50% at six months and 10% to 20% at one year [6]. Our clinical success following thrombolysis for a thrombosed AV access was 75%, in keeping with the reported literature. Our cohort also had equivalent steep attrition in clinical patency, reported in the literature at six months and one year [12]. Less than 10% of all AV accesses were clinically patent at three years in our cohort, regardless of whether they underwent further successful thrombolysis following a subsequent episode of AV access thrombosis. The high attrition rates following thrombolysis can be in part attributed to AV access age. Indeed, the median age of thrombosed AV fistulas receiving thrombolysis in our cohort was eight years, compared to AV grafts, which were under two years old. We did not find AV access survival following clinically successful thrombolysis differed by access type.

Twenty-one AV accesses presented with further AV access thrombosis and underwent a further episode of thrombolysis during the study period. A previous study found leg grafts to have higher levels of thrombosis than other access types and a higher requirement for interventions (1.3 angioplasties and 0.12 thrombolytic procedures per patient per year) [19]. This study found that AVGs were more likely to present with re-thrombosis and undergo further thrombolysis than AVFs and to present with re-thrombosis significantly earlier than AVFs. However, no differences were found in overall AV access survival between AVG and AVF, albeit there was a non-significant trend favouring repeat thrombolysis on AV access survival overall and in AVF alone.

Repeated thrombosis is likely to represent an AV access nearing the end of its life, and as such, successful repeat thrombolysis should allow the MDT time for planning alternative AV access, which should ideally be the formation of a new surgical fistula or AV graft.

A total of 90% of access thrombosis has been reported to be attributable to an underlying anatomic stenosis [20,21]. A total of 57% of our cohort had a preceding endovascular intervention for an underlying AV access stenosis, with no differences between AVFs and AVGs. 

All radiologically detected significant lesions were treated in patients undergoing fistuloplasty prior to presenting with thrombosis, regardless of their clinical significance. This strategy has proven ineffective at prolonging AV access survival, given that previous angioplasty was associated with no fistula survival advantage over those presenting de novo. We previously showed no benefit in radiological surveillance over and above clinical examination following fistula surgery and demonstrated a lower primary patency associated with more angioplasty in the surveillance group [22]. Two meta-analyses of access surveillance showed a reduction in the relative risk of access thrombosis but did not show a reduction in the risk of access loss [23,24]. A Cochrane review also concluded that there was no advantage of preemptive correction of a functioning AV stenosis that improved longevity or prevented access loss [9]. We therefore purposefully refrained from emphasizing radiologic primary and secondary patency rates, as these do not necessarily translate to functional patency. In addition to technical patency, there is a requirement for sufficient AV access flow to allow adequate haemodialysis. A patent AV access that doesn’t sustain this is no better than a thrombosed fistula, i.e., unfit for purpose, and so our study focused on clinical patency, i.e., the patient receiving adequate haemodialysis, rather than radiological patency, which may not necessarily translate into useable access.

It is our opinion that, prior to deciding upon whether to consider repeated thrombolysis, the MDT needs to establish what future access options exist and whether a repeated thrombosis represents AV access reaching the end of its life. We believe there is still some utility in attempting repeat thrombolysis in certain situations, such as in patients with limited future access options or those with AV grafts, as even a short-term benefit would delay dialysis catheter insertion and its subsequent risks to the patient. A total of 40% of our patients having successful thrombolysis were alive at 10 years, and so consideration of the patients’ comorbidities and fitness for thrombolysis and further surgical AV access should be made by the MDT, as a tunnelled catheter may well be the right access for high-risk patients with a thrombosed fistula or graft.

Our study is limited by its retrospective design, which does not allow for a control group comparison. It also meant we could not report on the proportion of thrombosed AV accesses that did not undergo thrombolysis at the first presentation.

Subgroup analysis between the different endovascular methods was also not possible due to the low numbers of patients having AngioJet™ and the heterogeneity of practice between different operators and often combinations of methods being used. We also did not report on thrombolysis-associated complications or time from presentation to thrombolysis and its effect on outcomes, although our institution previously reported no adverse outcomes with delays greater than 48 hours from presentation to thrombolysis [10].

## Conclusions

Endovascular strategies for dealing with thrombosed access are clinically important in allowing haemodialysis to continue in patients with end-stage renal failure. However, the clinical patency rates, while good in the short term, fail to be sustained in the long term. We propose that an episode of AV access thrombosis and successful thrombolysis should prompt a review of previous access-related interventions, age of AV access, and planning for alternative AV access options, be it new AV access formation, transplant, or long-term catheter placement.
